# Supplementary material for: EnzML: multi-label prediction of enzyme classes using InterPro signatures
Source: BMC Bioinformatics. 2012 Apr 25;13:61. doi: 10.1186/1471-2105-13-61 (PMC3483700; doi:10.1186/1471-2105-13-61)
Supplement: Addtional file 5 — The Java code to format the data files, evaluate and predict. The file enzml_java_code.tar.gz contains the Java code used to format database data to ARFF and XML formats, to execute cross and train-test (jackknife) evaluations and to record evaluation results to database. More information is included in the readme.txt file and the Javadoc files. The code can be used with a MySQL database. To use a different database software, other JDBC drivers might be required. [file 1471-2105-13-61-S5.gz › java_code/enzml2011/doc/uk/ac/ed/inf/taweka/mulan/learn/MulanLearner.html]

MulanLearner


---


|  |  |  |
| --- | --- | --- |
| **Overview** \| **Package** \| **Class** \| **Use** \| **Tree** \| **Deprecated** \| **Index** \| **Help** | |  |
| **PREV CLASS**   **NEXT CLASS** | **FRAMES**    **NO FRAMES**     **All Classes** |  |
| SUMMARY: NESTED \| FIELD \| CONSTR \| METHOD | DETAIL: FIELD \| CONSTR \| METHOD |  |


---


## uk.ac.ed.inf.taweka.mulan.learn Class MulanLearner

```
java.lang.Object
  uk.ac.ed.inf.taweka.mulan.learn.MulanLearner
```

---

``` public class MulanLearner extends java.lang.Object ```

Containing a Mulan learner and its name, that is: the name of the
MultiLabelLearnerBase plus the name of the algorithm within

**Version:**
:   1 Sep 2010

**Author:**
:   Luna De Ferrari luna.deferrari-at-ed.ac.uk

---

**Constructor Summary**

`MulanLearner(java.lang.String learnerBaseType,
java.lang.String learnerType,
mulan.classifier.MultiLabelLearnerBase learner)`


| **Method Summary** | |
| --- | --- |
| `mulan.classifier.MultiLabelLearnerBase` | `getLearner()` |
| `java.lang.String` | `getLearnerBaseType()` |
| `java.lang.String` | `getLearnerFullName()`             Get the full type of a learner: multi-label learner base + algorithm within |
| `static java.lang.String` | `getLearnerName(java.lang.Object learner)` |
| `java.lang.String` | `getLearnerType()` |

**Methods inherited from class java.lang.Object**

`equals, getClass, hashCode, notify, notifyAll, toString, wait, wait, wait`

**Constructor Detail**

### MulanLearner

```
public MulanLearner(java.lang.String learnerBaseType,
                    java.lang.String learnerType,
                    mulan.classifier.MultiLabelLearnerBase learner)
```


**Method Detail**

### getLearner

```
public mulan.classifier.MultiLabelLearnerBase getLearner()
```

---


### getLearnerBaseType

```
public java.lang.String getLearnerBaseType()
```

---


### getLearnerFullName

```
public java.lang.String getLearnerFullName()
```

:   Get the full type of a learner: multi-label learner base + algorithm
    within

---


### getLearnerType

```
public java.lang.String getLearnerType()
```

---


### getLearnerName

```
public static java.lang.String getLearnerName(java.lang.Object learner)
```


---


|  |  |  |
| --- | --- | --- |
| **Overview** \| **Package** \| **Class** \| **Use** \| **Tree** \| **Deprecated** \| **Index** \| **Help** | |  |
| **PREV CLASS**   **NEXT CLASS** | **FRAMES**    **NO FRAMES**     **All Classes** |  |
| SUMMARY: NESTED \| FIELD \| CONSTR \| METHOD | DETAIL: FIELD \| CONSTR \| METHOD |  |


---
